# Marshall Hall’s Ready Method

**Published:** 1859-04

**Authors:** George V. Ewing

**Affiliations:** Rock Run, Illinois


ARTICLE IV.

### MARSHALL HALL'S *READY METHOD.*

BY GEORGE V. EWING, M. D., OF ROCK RUN, ILLINOIS.

DR. N. S. DAVIS:

DEAR SIR,—In visiting your city last December, I did intend to make a request of you, but finding your time to be very precious, I thought best not to intrude upon you at that time. But now, by your permission, I will make the request. It is this, that you will publish in the *Journal*, for the benefit of its numerous readers, the *Ready Method* in Asphyxia, of Sir Marshall Hall, as it appears in the *London Lancet*, vol. i., 1857, page 148.

I am not aware that the method has ever been published in the *Journal*, and, I believe, I never have seen it in any of the medical journals to which I have had access, save the *London Lancet.* Hence it is that I make the request.

The method, aside from its importance in the treatment of drowned persons, possesses superior merits, and may be brought into almost daily operation in obstetrical practice. In my own experience I have had occasion to resort to it in a number of still-born infants, and have been highly gratified with the result in every case; and I am satisfied that some of the cases could not have been resuscitated by any other means.

This may be regarded as one of the great improvements of the day in our noble profession, and the name of its late author will, doubtless, be considered a sufficient guarantee for its superior merits. But permit me to add my testimony in its favor, by reporting a single case from the number of those who have had the benefit of the method in my practice.

September 19th, 1858, I was called in haste to visit Mrs. H., who was in labor with her first child. On my arrival, I found that the membranes had been ruptured and the liquor amnii discharged some time previous to my arrival. Pains were at this time regular, and moderately severe, occurring at intervals of about every ten minutes. Upon making an examination, I found that I had a case of foot presentation to treat. In five pains after my attendance, the lower extremities and chest were born; after this the pains became very feeble and irregular. As there was great pressure upon the umbilical cord, causing the circulation entirely to cease, I proceeded to terminate delivery immediately; this, however, occupied considerable time, owing to great difficulty in bringing down the arms. The delivery of the head also proved to be very tedious, but by persevering, with all the force that was admissible in the case, delivery was finally accomplished. The time which elapsed from the last motion (which was very feeble) till delivery was terminated, was about thirty minutes.

The child, when born, was, to all appearance, lifeless. The cord was ligated and cut immediately, and as the mother did not require any immediate assistance, I turned my attention to the child to see what could be done. I first cleared its mouth and throat of mucus, which was rather abundant; then applied the method unceasingly for fifteen minutes, at the end of which time I had the pleasure of seeing my little patient give slight evidence of returning animation. I continued the method for twenty minutes longer and respiration was fully established, though the child was very feeble. I then enveloped it in some warm flannels, turned it on its side, and handed it to the nurse, gave the requisite attention to the mother, and in an hour left the house, with directions not to wash and dress the child for at least six hours. On my visit next day, twenty-four hours *post part.*, I found my little patient crying lustily. The child has continued to do well ever since, and at this date may well be considered as a fine specimen of *a sucker*, and the parents exalt the Ready Method of Sir Marshall Hall. Yours, etc.,

GEO. V. EWING, M.D.

ROCK RUN, ILL., March 30th, 1859.

[We republish, with pleasure, Marshall Hall's *Ready Method*, as furnished to the present editor by himself, although it was once inserted in this *Journal*. In so doing we would add, that this method seems to us imperfect in many respects, particularly in what relates to artificial respiration and the restoration of a proper temperature.—ED.]

THE READY METHOD IN SUSPENDED RESPIRATION FROM

### DROWNING, ETC.

By Marshall Hall, M.D., F.R.S., of the Institute of France, etc., etc.

1. Treat the patient *instantly*, *on the spot*, *in the open air*, *exposing* the face and chest to the *breeze* (except in severe weather).

*I.—To Clear the Throat—*

2. Place the patient gently on the face, with one *wrist* under the forehead;

[*all fluids and the tongue itself then fall forwards, leaving the entrance into the windpipe* FREE.]

If there be breathing—wait and *watch;* if not, or if it *fail*,—

*II.—To Excite Respiration—*

3. Turn the patient well and *instantly* on his side, and—

4. Excite the nostrils, the throat, etc., and dash cold water on the face previously rubbed warm.

If there be no success, *lose not a moment*, but *instantly*—

*III.—To Imitate Respiration—*

5. Replace the patient on his face, *raising* and supporting the chest *well* on a folded coat or other article of dress;

6. Turn the body very *gently on the side and a little beyond*, and then *briskly* on the face, alternately; repeating these measures deliberately, efficiently, and perseveringly *fifteen* times in the minute, occasionally *varying* the *side;*

[*when the patient reposes on the chest, this cavity is compressed by the weight of the body, and expiration takes place; when he is turned on the side, this pressure is removed, and inspiration occurs.*]

7. When the *prone* position is resumed, *make* equable but efficient *pressure*, with brisk movement, *along* the back of the *chest; removing* it immediately before rotation on the side;

[*the first measure augments the expiration, the second commences inspiration.*]

*** The result is—Respiration;—and, if not too late,—Life!

*IV.—To Induce Circulation and Warmth—*

8. Meantime rub the limbs *upwards*, with *firm grasping pressure* and with *energy*, using handkerchiefs, etc.;

[*by this measure the blood is propelled along the veins towards the heart.*]

9. Let the limbs be thus warmed and dried, and then clothed, each bystander supplying a coat, a waistcoat, etc.

10. *Avoid the continuous warm-bath, and the position on or inclined to the back.*

